# Mitochondrial quality control in lung diseases: current research and future directions

**DOI:** 10.3389/fphys.2023.1236651

**Published:** 2023-07-19

**Authors:** Jiliu Liu, Junyi Wang, Anying Xiong, Lei Zhang, Yi Zhang, Yao Liu, Ying Xiong, Guoping Li, Xiang He

**Affiliations:** ^1^ Laboratory of Allergy and Precision Medicine, School of Medicine, Southwest Jiaotong University, Chengdu Institute of Respiratory Health, The Third People’s Hospital of Chengdu, Affiliated Hospital of Southwest Jiaotong University, Chengdu, China; ^2^ Department of Pulmonary and Critical Care Medicine, Chengdu Third People’s Hospital Branch of National Clinical Research Center for Respiratory Disease, Affiliated Hospital of ChongQing Medical University, Chengdu, China; ^3^ Department of Pulmonary and Critical Care Medicine, Sichuan Friendship Hospital, Chengdu, China

**Keywords:** mitochondrial quality control, chronic obstructive pulmonary disease, lung cancer, idiopathic pulmonary fibrosis, therapeutic approaches

## Abstract

Lung diseases are a major global health problem, affecting millions of people worldwide. Recent research has highlighted the critical role that mitochondrial quality control plays in respiratory-related diseases, including chronic obstructive pulmonary disease (COPD), lung cancer, and idiopathic pulmonary fibrosis (IPF). In this review, we summarize recent findings on the involvement of mitochondrial quality control in these diseases and discuss potential therapeutic strategies. Mitochondria are essential organelles for energy production and other cellular processes, and their dysfunction is associated with various diseases. The quality control of mitochondria involves a complex system of pathways, including mitophagy, mitochondrial biogenesis, fusion/fission dynamics, and regulation of gene expression. In COPD and lung cancer, mitochondrial quality control is often involved in disease development by influencing oxidative stress and apoptosis. In IPF, it appears to be involved in the disease process by participating in the cellular senescence process. Mitochondrial quality control is a promising target for therapeutic interventions in lung diseases. However, there are conflicting reports on different pathological processes, such as the role of mitochondrial autophagy in lung cancer, which pose difficulties in the study of targeted mitochondrial quality control drugs. Additionally, there seems to be a delicate balance between the mitochondrial quality control processes in the physiological state. Emerging evidence suggests that molecules such as PTEN-induced putative kinase 1 (PINK1), parkin RBR E3 ubiquitin protein ligase (PRKN), dynamin-related protein 1 (DRP1), and peroxisome proliferator-activated receptor gamma coactivator 1-alpha (PGC1-α), as well as the signaling pathways they affect, play an important role in respiratory-related diseases. Targeting these molecules and pathways could contribute to the development of effective treatments for lung diseases. In conclusion, the involvement of mitochondrial quality control in lung diseases presents a promising new avenue for disease treatment. Further research is needed to better understand the complex mechanisms involved in the pathogenesis of respiratory diseases and to develop targeted therapies that could improve clinical outcomes.

## 1 Introduction

The mitochondrion is a unique organelle that carries its own genetic material, known as mitochondrial DNA (mtDNA) and functions as a semi-autonomous cell. Although it was previously believed that mitochondria were primarily responsible for intracellular energy metabolism, modern scientific research has demonstrated they play a wide range of other vital roles within the cell. Besides producing ATP through oxidative phosphorylation, mitochondria are also involved in cellular processes such as calcium metabolism, aging, apoptosis, programmed death (Picard and Shirihai, 2022). Recent studies have shown that mitochondria are also involved in intercellular communication. Specifically, scientists have discovered that mitochondria in mouse neuronal axon cells are secreted outside the cell, taken up by neighboring glial cells, and eventually destroyed by lysosomes (Davis et al., 2014). Mitochondrial quality control is crucial for maintaining mitochondrial and intracellular homeostasis, and it usually involves three primary processes: mitochondrial biogenesis, mitochondrial dynamics (primarily fusion and fission), and mitophagy. Along with mitochondrial biogenesis, dynamics, and mitophagy, researchers have included mitochondrial regulation of mtDNA, mitochondrial unfolded protein response (UPRMT), and mitochondrial contact with other organelles such as the endoplasmic reticulum into the idea of mitochondrial quality control (Ng et al., 2021). Various forms of cellular stress can induce mitochondrial quality control, including reactive oxygen species (ROS), environmental pollutants, and ionizing radiation. These stresses can cause normal mitochondria to deteriorate, resulting in mtDNA mutations, changes to the mitochondrial membrane potential, and issues with calcium metabolism. When this happens, the mitochondrial quality control system initiates a response, which includes eliminating unrepairable mitochondria through fission and mitophagy, maintaining mitochondria function through fusion, and filling the energy metabolism gap via mitochondrial biogenesis. These mechanisms work together to maintain optimal mitochondrial and intracellular homeostasis.

The mitochondrial quality control system plays a vital role in maintaining mitochondrial and cellular homeostasis and is critical in protecting against oxidative stress, which can lead to disorders associated with cellular senescence in respiratory diseases. Dysregulation of mitochondrial biogenesis, mitophagy, and dynamics can lead to chronic inflammation, fibrosis, and other pathophysiological changes commonly observed in respiratory conditions like COPD, IPF and lung cancer. A better understanding of the mitochondrial quality control system’s role in respiratory diseases will help develop new treatments to slow or stop disease progression.

## 2 Mitochondrial quality control

### 2.1 Mitophagy

Mitophagy is a type of macroautophagy that specifically targets damaged or dysfunctional mitochondria for degradation in order to maintain cellular integrity and health. This process is essential for removing old, damaged or excess mitochondria, which can accumulate and lead to cell dysfunction or death if not properly disposed of. By selectively targeting and eliminating these damaged mitochondria, mitophagy helps to ensure the quality and functionality of the remaining mitochondria (Ashrafi and Schwarz, 2013). Previous research on mitophagy has identified two primary directions: the PINK1/PRKN-dependent and PINK1/PRKN-independent mitophagy (Choi et al., 2013). PINK1 is a protein kinase located on the surface of mitochondria. Normally, PINK1 is cleaved by presenilin associated rhomboid like (PARL) and maintained at low levels in healthy mitochondria. However, in damaged or injured mitochondria, PINK1 is not cleared and instead accumulates on the outer mitochondrial membrane, where it serves as a marker for damaged mitochondria. This accumulation of PINK1 triggers a series of events that are critical for mitochondrial quality control. One of the key actions of accumulated PINK1 is the phosphorylation of the Ser65 site on ubiquitin molecules. Phosphorylated ubiquitin acts as a signal to recruit PRKN, from the cytoplasm to the affected mitochondria. Once recruited, PRKN interacts with the phosphorylated ubiquitin on the outer mitochondrial membrane (OMM). This interaction triggers a series of events that ultimately lead to the selective degradation of the damaged mitochondria through mitophagy (Wang et al., 2019; Pan et al., 2020; Di Rienzo et al., 2022). Furthermore, it has been found that PTEN-L, a novel member of the PTEN family, has inhibitory effects on PRKN mitochondrial translocation, phosphorylation, and tends to adopt a closed conformation. Importantly, PTEN-L also reduces the overall level of intracellular phosphorylation, mainly by decreasing the level of intracellular phosphorylated ubiquitin (pSer65-Ub) (Wang et al., 2018). Another key player in the regulation of mitophagy is the deubiquitinating enzyme. Studies have revealed that the deubiquitinating enzyme ubiquitin specific peptidase 30 (USP30) negatively regulates mitophagy by mediating PRKN deubiquitination (Gersch et al., 2017). Niu et al. (2020) unveiled for the first time that another deubiquitinating enzyme, ubiquitin specific peptidase 33 (USP33), localizes to the outer mitochondrial membrane and directly interacts with the E3 ubiquitin ligase PRKN. They demonstrated that USP33 can remove various ubiquitin chains, including K6, K11, K48, and K63, which are bound to PRKN. Apart from the PTEN family members, researchers have also discovered other proteins involved in regulation of mitophagy. One such protein is Sam50, also known as SAMM50 or OMP85. Knockdown of Sam50 in cells has been shown to enhance mitophagy. Interestingly, although Sam depletion leads to a decrease in mitochondrial mass and subsequent aggregation of PINK1, this mechanism does not affect mitochondria to the extent of mtDNA. Hence, it has been confirmed that PINK1-mediated mitophagy triggered by Sam50 depletion actually functions as a protective mechanism for mtDNA (Jian et al., 2018).

Mitophagy can occur through both the PINK1/PRKN-dependent pathway and alternative, PINK1/PRKN-independent pathways. Certain receptor proteins for mitophagy have been identified on the OMM that can directly bind to LC3-positive autophagosomes and initiate mitophagy. These receptors include BCL2 interacting protein 3 (BNIP3) and BCL2 interacting protein 3 like (BNIP3L/NIX). BNIP3 and BNIP3L/NIX are HIF-1 target genes that possess LC3-interacting regions (LIRs) and can attract LC3 autophagosomes to induce mitophagy (Zhang et al., 2019). Another recently discovered autophagy receptor protein is FUN14 domain containing protein (FUNDC1), which is involved in mitophagy induction. Under hypoxic conditions, FUNDC1 undergoes dephosphorylation and enhances its interaction with LC3D, promoting mitophagy (Liu et al., 2012). Both of these PINK1/PRKN-independent mitophagy pathways are induced by altered cellular environments, particularly hypoxia or increased levels of ROS. As for negative feedback regulation of mitophagy, BCL2 like 1 (BCL2L) has been found to inhibit mitophagy by preventing the dephosphorylation of FUNDC1 (Wu et al., 2014). In *in vitro* experiments, researchers have observed that under hypoxic or starvation conditions, mitochondria can elongate or fuse with each other to form larger mitochondria. This pattern of mitochondria exhibits resistance to mitophagy, ensuring the maintenance of cellular energy supply during hypoxic and starvation conditions. However, it is important to note that overall mitophagy levels are upregulated in hypoxic or starvation environments (Rambold et al., 2011).

### 2.2 Mitochondrial dynamics

Mitochondrial dynamics refers to the process of continuous changes in mitochondrial morphology, distribution, and function through mitochondrial fusion, fission, and trafficking. Mitochondrial fusion is the process by which two or more mitochondria merge into one, while fission is the opposite process, where a mitochondrion divides into two or more smaller ones. The balance between fusion and fission regulates the number, size, and distribution of mitochondria within the cell, and any dysregulation can lead to aberrant mitochondrial function and contribute to disease pathogenesis.

DRP1 and its inhibitors Mdivi-1, as well as Fission1 (FIS1) and mitochondrial fission factor (MFF), are key molecules involved in mitochondrial fission. When mitochondria are damaged, DRP1 is recruited to the OMM, but it does not directly bind to the OMM. Instead, it forms a complex with the adapter proteins FIS1 and MFF on the OMM. Through the hydrolysis of GTP and the contraction of the DRP1 helix structure, the integrity of the OMM is disrupted, initiating the process of fission. Recent research has revealed that mitochondrial fission involves the coordinated actions of several organelles. It has been found that mitochondrial fission occurs at the contact points between the OMM and endoplasmic reticulum (ER) tubules, which are determined prior to the recruitment of DRP1 (Friedman et al., 2011). Furthermore, two proteins called inverted formin 2 (INF2) and Spire1C (a splice variant of Spire1) are present at these contact points and bind to actin, causing premature contraction of mitochondria. This premature contraction contributes to the recruitment of DRP1. *In vitro* experiments have confirmed that knockdown of INF2 and Spire1C lead to elongated mitochondria and reduced binding of DRP1 to mitochondria (Korobova et al., 2013; Manor et al., 2015). Interestingly, studies have shown that the choice of mitochondrial fission sites is closely related to the state of the mitochondria (Kleele et al., 2021). Researchers studying mitochondrial fission in mouse cardiomyocytes discovered two distinct types of fission: midzone fission and peripheral fission. Midzone fission occurs at the middle of the mitochondria and involves precontraction of the ER with actin intervention, as well as the involvement of MFF. On the other hand, peripheral fission occurs around the mitochondria and does not exhibit the same precontraction and MFF involvement. The selection of fission type is determined by the condition of the mitochondria, with midzone division occurring in healthier mitochondria and peripheral division occurring in damaged mitochondria, which are subsequently cleared through mitophagy. Research on the mechanism of fission in the inner mitochondrial membrane (IMM) is still in an exploratory stage, but it is closely related to ER tubules. Evidence suggests that ER tubules assist in the division of mtDNA into daughter mitochondria, which is the first step in mitochondrial fission occurring in the IMM (Lewis et al., 2016). Additionally, INF2 at the ER tubule-mitochondria binding site has been found to mediate the entry of Ca^2+^ into mitochondria, leading to a change in mitochondrial membrane potential and contraction of the inner mitochondrial compartment (Cho et al., 2017).

Mitochondrial fusion-related proteins play a crucial role in maintaining mitochondrial integrity and function. These proteins include OPA1 mitochondrial dynamin like GTPase (OPA1), which regulates inner IMM fusion, and mitofusin 1 and mitofusin 2 (MFN1 and MFN2), which regulate OMM fusion. Mitochondrial fusion is an essential physiological event in eukaryotic cells as it allows for the exchange of lipids and proteins, fusion of mtDNA, and shared resistance to mitophagy (Giacomello et al., 2020). The fusion process begins with the docking of two MFN1 molecules, leading to a conformational change (likely through the binding of the GTPase structural domain) that drives GTP hydrolysis by MFN1. This facilitates OMM fusion. The role of MFN2 in fusion is also significant, although there are conflicting reports regarding its specific function (de Brito and Scorrano, 2008; Filadi et al., 2015; Naon et al., 2016). Knocking out the MFN2 gene has been shown to result in enlarged mitochondria, suggesting its involvement in fusion. Some studies suggest that MFN2 acts as a bridge between the ER and mitochondria, facilitating mitochondrial calcium uptake and regulation of mitochondrial membrane potential. On the other hand, other studies propose that MFN2 acts to prevent excessive proximity between the ER and mitochondria, thereby preventing cytotoxicity. Further research is needed to fully elucidate the precise role of MFN2 in fusion.

In addition to mitochondrial fission and fusion, researchers are exploring additional aspects of mitochondrial dynamics. Desai et al. (2020) discovered a retrograde communication pathway between mitochondria and the nucleus called mitochondria-to-nucleus retrograde signaling (MRR). This communication is mediated by translocator protein (TSPO) and involves the formation of contact sites between mitochondria and the nucleus. Through this communication, mitochondria actively relay information to the nucleus and play a crucial role in the cell’s response to stress, ultimately contributing to the extension of the cell’s lifespan. Notably, mitochondria establish contact sites with various organelles, including the ER, lysosomes, and golgi apparatus, enabling communication with these organelles. This behavior suggests a higher level of coordination and “intelligence” compared to other organelles, supporting the hypothesis of mitochondrial origin through endosymbiosis. Furthermore, Jiao et al. (2021) reported a novel mechanism of mitochondrial quality control known as “Mitocytosis.” This process involves the expulsion of damaged mitochondria from the cell via cytokinesis, facilitated by migrosomes.

### 2.3 Mitochondrial biogenesis

Mitochondrial biogenesis is the process of generating new mitochondria within cells. It is a complex process that involves the coordinated expression of multiple genes and transcription factors, as well as the replication and assembly of mitochondrial DNA and proteins. PGC-1 is the central molecule in mitochondrial biogenesis (Scarpulla et al., 2012). The members of the PGC-1 family include PGC-1α, PGC-1β, and PRC (PGC-1α-related coactivator). In mammals, PGC-1α plays a primary role in regulating mitochondrial biogenesis (Ding et al., 2021). Activation of PGC-1α leads to the expression of nuclear respiratory factor NRF1/2, which is sensitive to intracellular redox state and regulates cellular redox (Cui et al., 2021). Mitochondrial transcription factor (including TFAM, TFB1M, TFB2M) then enters the mitochondria and binds to mtDNA, activating RNA polymerase and initiating transcription. The orphan nuclear receptors ERRS (ERRα, ERRβ, ERRγ) regulate PGC1 activity in the PGC1 pathway. Although PGC-1α and PGC-1β share similar structure and function, studies have shown that PGC-1α expression is associated with ERRγ, while PGC-1β is mainly associated with other ERR family members (Shao et al., 2010; Sakamoto et al., 2022). Additionally, PGC-1β appears to play a specific role in certain pathological processes, as a study on hepatocellular carcinoma found downregulation of PGC-1β expression in drug-resistant cancer cells (Xu J. et al., 2021). The regulation of mitochondrial biogenesis generally begins with AMP-activated protein kinase (AMPK), a key factor in sensing energy status (Hardie et al., 2012). AMPK is activated by phosphorylation of AMPL by LKB1 when intracellular AMP/ATP levels rise, indicating low ATP levels and energy stress. Activation of AMPK regulates mitochondrial biogenesis and relieves intracellular energy stress. Zhang et al. (2013) identified the AXIN-AMPK-LKB1 complex as a key factor linking AMP/ATP levels to AMPK activation. Importantly, excessive mitochondria lead to increased ROS, and NADPH Oxidase 4 (NOX4) counter-regulates mitochondrial biogenesis by inhibiting the survival of TFAM. This indirectly affects the cell’s bioenergetic reserve. Studies on NOX4 have also revealed that its mediation of mitochondrial biogenesis in lung fibroblasts appears to be independent of PGC-1α, as it can directly regulate NRF1 without involving PGC-1α (Bernard et al., 2017). Recent advancements in understanding mitochondrial biogenesis have identified new molecules and pathways. Masahiro Morita et al. demonstrated that the mechanistic target of rapamycin complex 1 (mTORC1) controls mitochondrial activity and biogenesis by repressing eukaryotic translation initiation factor 4E (eIF4E)-binding protein (4E-BP), which selectively promotes translation of nuclear-encoded mitochondria-associated mRNA (Morita et al., 2013). It was discovered that mechanistic target of rapamycin (mTOR) also plays a key role in mitochondrial dynamics, influencing mitochondrial division through control of mitochondrial fission process 1 (MTFP1) (Morita et al., 2017). Importantly, mTOR activation is common in cancer and may be related to the high energy demand in cancer cells (Mossmann et al., 2018). Studying the mTOR pathway’s involvement in mitochondrial biogenesis is expected to reveal new targets for cancer therapy.

## 3 COPD

**FIGURE 1:**
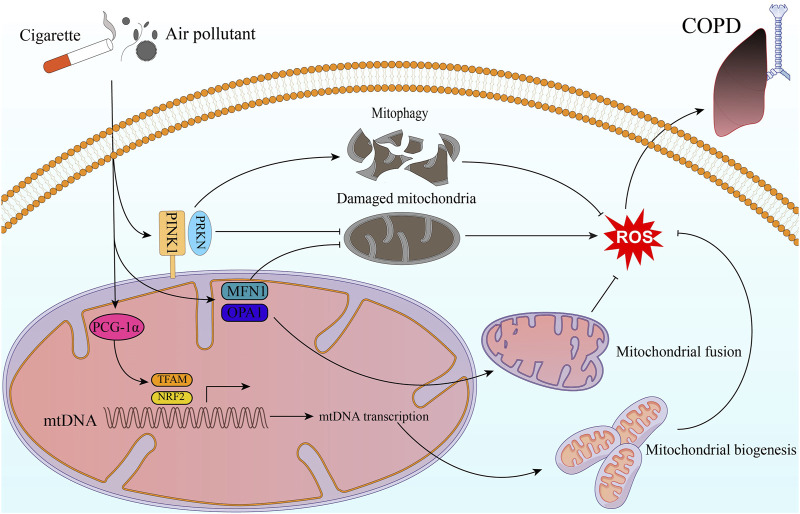
Mitochondrial quality control in COPD. Exogenous stimulants such as cigarette smoke and air pollutants impact ROS production through molecules related to mitochondrial quality control. This, in turn, contributes to the development and progression of COPD. The stimulatory factors can activate the PINK1/PRKN pathway, and then helps inhibit intracellular ROS production through mitophagy and clearing out damaged mitochondria. Additionally, PGC-1α activates NRF2 and TFAM pathways, leading to mitochondrial biogenesis. This process results in the generation of new, healthy mitochondria that are less prone to ROS production. Moreover, by promoting mitochondrial fusion and repairing damaged mitochondria, MFN1 and OPA1 contribute to maintaining mitochondrial function and reducing oxidative stress.

The World Health Organization (WHO) has reported that COPD caused 3.22 million deaths worldwide in 2019, which represents an increase of 17.5% from 2007 to 2017, the major burden of COPD deaths occurs in Latin America, sub-Saharan Africa, India, Southeast Asia, and China (GBD, 2019 Risk Factors Collaborators, 2020). This highlights the significant burden of this disease and the urgent need for effective interventions to reduce its impact on global health. Several lines of evidence suggest that impaired mitochondrial quality control may be a critical contributor to the development and progression of COPD (Figure 1).

### 3.1 Mitophagy in COPD

In COPD patients, there is a deficiency in mitophagy, leading to the accumulation of aberrant mitochondria within cells. These aberrant mitochondria can produce increased levels of reactive oxygen species and trigger an inflammatory response, contributing to the pathogenesis of COPD. PRKN, a key regulator of mitophagy, is downregulated in lung epithelial cells of COPD patients compared to non-COPD controls. The downregulation of PRKN in lung epithelial cells of COPD patients may contribute to the deficiency in mitophagy observed in these patients, leading to the accumulation of aberrant mitochondria and increased oxidative stress within cells (Ito et al., 2015). PINK1 and PRKN, two proteins involved in mitophagy, have shown interesting effects in the context of COPD and cigarette smoke (CS) exposure. *In vitro* experiments have suggested that even low levels of PINK1 expression can be compensated by increased PRKN expression, leading to induced mitophagy and reduction of cellular ROS stress during CS exposure, a major risk factor for COPD (Araya et al., 2019). However, the specific role of mitophagy in COPD is still not fully understood, and there are differing opinions on whether mitochondrial autophagy is protective or detrimental in COPD patients. Mizumura et al. (2014) found that inhibition of mitophagy reduced CS-induced mitochondrial dysfunction and cell death. Additionally, Pink1 (−/−) mice were protected against mitochondrial dysfunction, airspace enlargement, and disruption of mucociliary clearance during CS exposure. Although PINK1 and PARK2 act in the same direction in mitophagy, their role in other processes, such as apoptosis and necrosis, remains to be explored. Skeletal muscle dysfunction is a common comorbidity in COPD patients, with a reported frequency of up to 21.6%. This loss of muscle mass and strength can contribute to exercise intolerance, reduced quality of life, and increased mortality in COPD patients (Benz et al., 2019). Mitochondrial dysfunction and impaired mitophagy have been implicated in the development of skeletal muscle dysfunction in COPD patients. A study by Akihiko Ito et al. found that CS exposure can contribute to myotubular atrophy in skeletal muscle cells of COPD patients. It suggested that this may be due to a decrease in the expression level of PRKN, a key mediator of mitophagy, leading to insufficient mitochondrial clearance and the accumulation of ROS (Ito et al., 2022).

### 3.2 Mitochondrial dynamics in COPD

Mitochondrial morphology and dynamics are altered in the lung cells of COPD patients. In emphysema patients, the researchers found that the mitochondrial morphology of alveolar type 2 cells was abnormal, with impaired fission and fusion processes compared to normal human alveolar type 2 cells (Kosmider et al., 2019). mtDNA is more prone to damage under stress due to the absence of protective histones present in nuclear DNA (Fontana and Gahlon, 2020). When mtDNA is depleted or damaged, mitochondria tend to upregulate the expression of fusion-related proteins such as MFN1 and OPA1 in order to compensate for their metabolic deficiencies and maintain cell function (Yang et al., 2015). In studies of human alveolar epithelial cells, treatment with nontoxic doses of CS exposure was found to induce mitochondrial hyperfusion and increase the expression of mitochondrial fusion-related proteins such as MFN1. This led to enhanced mitochondrial function and increased cellular respiration, suggesting that CS exposure may have therapeutic potential for the treatment of lung diseases such as COPD and emphysema (Ballweg et al., 2014). However, *in vitro* experiments have shown that exposure to high concentrations of CS exposure can induce mitochondrial fragmentation in BESA-2B cells (Hoffmann et al., 2013). Similarly, mitochondrial fragmentation has been observed in the lung epithelial cells of COPD patients who smoke (Hara et al., 2013). In addition to inducing mitochondrial fragmentation, higher doses and longer exposures to CS result in a greater decrease in mitochondrial cristae. Interestingly, there is a paradoxical increase in OPA1 expression in response to CS stimulation, which is normally associated with an increase in cristae number (Varanita et al., 2015). This suggests that the relationship between OPA1 expression and cristae morphology may be more complex. Indeed, studies have shown that exposure to atmospheric pollutants such as fine particulate matter (PM2.5) can also lead to an increase in OPA1 expression accompanied by a loss of mitochondrial cristae (Li et al., 2015). Indeed, previous research has provided evidence for the significant connection between ER and mitochondrial function. It has been found that MFN2 plays a role in linking mitochondria to the ER during ER stress (Bueno et al., 2015). This link may be compromised due to impaired Ca^2+^ communication between the ER and mitochondria. Subsequent studies have also supported the notion that ER stress can lead to impaired mitochondrial fusion, which subsequently affects mitochondrial function in alveolar epithelial cells (Bravo et al., 2011). While the precise relationship between ER stress and mitochondrial dysfunction in the context of COPD is yet to be fully demonstrated, these findings suggest that the communication between mitochondria and the ER may influence mitochondrial metabolic function in COPD.

### 3.3 Mitochondrial biogenesis in COPD

Studies have shown that COPD patients have reduced mitochondrial counts in their lung tissue compared to healthy individuals, likely due to increased cellular death and impaired mitochondrial biogenesis (Kirkham and Barnes, 2013). PCG-1α is a transcriptional coactivator known to play an important role in regulating mitochondrial biogenesis and oxidative metabolism. Studies have shown that PCG-1α levels are elevated in the lung tissue of patients with mild COPD (Li et al., 2010). This increase in PCG-1α expression is thought to be due to intracellular oxidative stress, which is a hallmark of COPD pathogenesis. However, in severe COPD or under the effect of severe CS stimuli, the regulation of PCG-1α becomes dysregulated. Studies have shown that in severe COPD patients, there is a downregulation of PCG-1α expression in lung tissue, which could contribute to mitochondrial dysfunction and impaired oxidative metabolism. This may be due to a number of factors, including increased inflammation, oxidative stress, and DNA damage in lung tissue. NRF2 is a transcription factor, sensing cellular redox state and regulating the expression of antioxidant and cytoprotective genes. Studies have shown that NRF2 levels are reduced in the lung tissue of patients with COPD. It reported that pharmacological activation of NRF2 can improve lung function, reduce inflammation, and protect against oxidative damage in models of COPD (Bocci and Valacchi, 2015). AMPK (adenosine monophosphate-activated protein kinase) is a crucial regulator of cellular energy homeostasis, and it has been shown to regulate mitochondrial biogenesis and function. Although, there have been limited studies investigating the role of AMPK in regulating mitochondrial biogenesis in COPD, studies have confirmed that AMPK expression is upregulated in response to cigarette smoke exposure in both human and animal models (Tang et al., 2011).

## 4 Lung cancer

**FIGURE 2:**
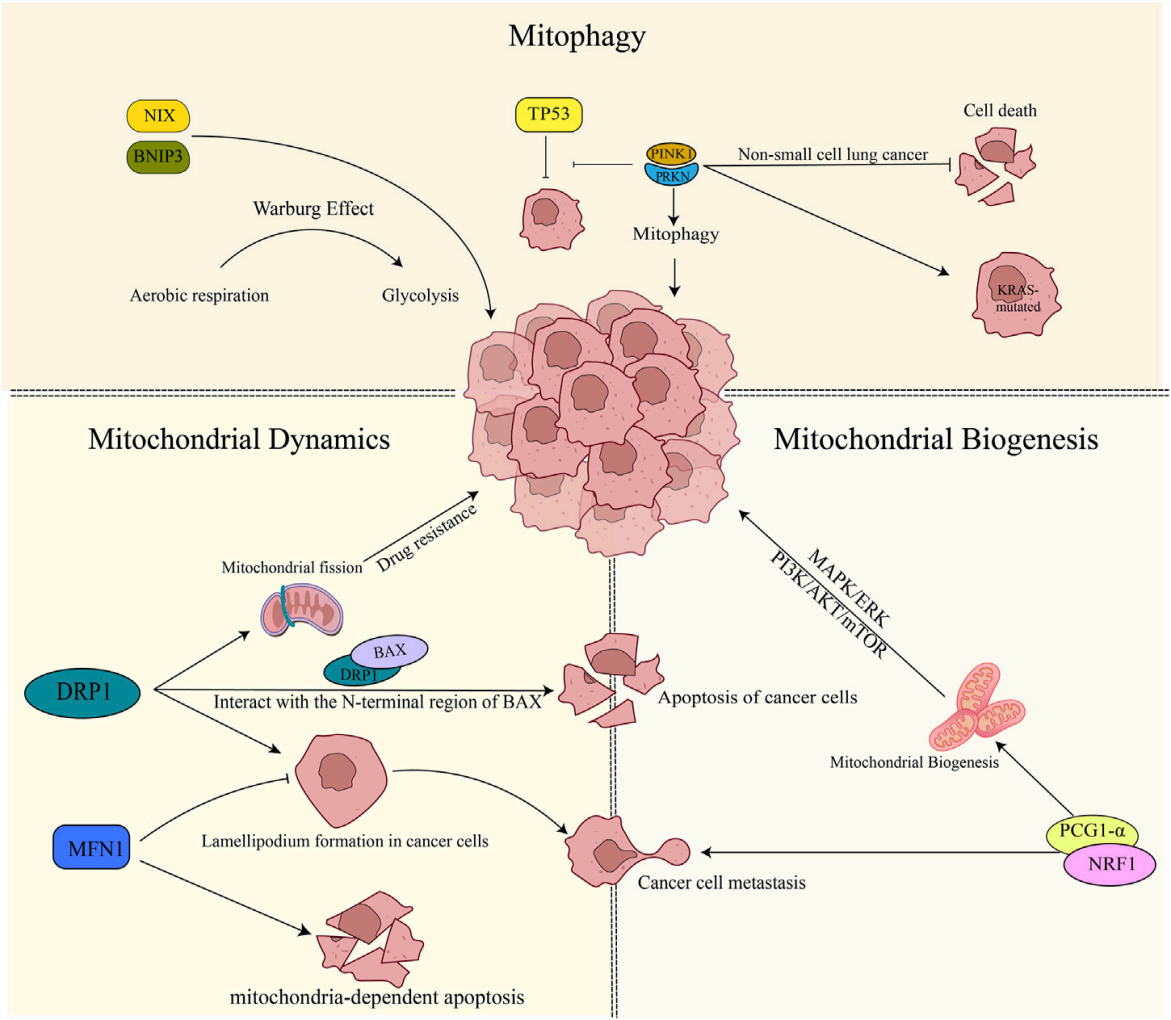
Mitochondrial quality control in lung cancer. 1) PINK1/PRKN plays a role in suppressing apoptosis in non-small cell lung cancer cells. Additionally, it promotes the mutation of the KRAS gene and inhibits the oncogenic process mediated by TP53. On the other hand, BNIP3/NIX facilitates tumor cell growth by promoting the Warburg effect. 2) DRP1 increases drug resistance to tumor cells by boosting mitochondrial fusion. It also physically interacts with the N-terminal region of BAX, promoting apoptosis. Inhibition of DRP1 or overexpression of MFN1 reduces the formation of cell lamellipodia, thereby inhibiting cancer cell migration. Furthermore, the expression of MFN1 contributes to mitochondria-dependent apoptosis. 3) Mitochondrial biogenesis plays a role in promoting tumor development through the activation of the MAPK/ERK pathway, PI3K/AKT/mTOR signaling, and the involvement of PGC-1α. In particular, PGC-1α expression contributes to tumor cell metastasis.

Lung cancer accounts for around 11.6% of all cancer cases and is the most prevalent form of cancer globally. Moreover, it is the leading cause of cancer deaths worldwide, responsible for approximately 18.4% of all cancer-related fatalities. Lung cancer affects men more frequently than women and is the number one cause of cancer deaths in men worldwide. Nevertheless, it is also the second leading cause of cancer deaths in women, following breast cancer (Bray et al., 2018). Targeted therapy and immunotherapy have emerged as promising treatment approaches for lung cancer. However, their clinical effectiveness has been limited by several factors (Hirsch et al., 2017). Mitochondria are essential for cellular energy production, metabolism, and signaling. Alterations in mitochondrial quality control may be involved in the development and progression of lung cancer (Xu et al., 2013; Naik et al., 2019) (Figure 2).

### 4.1 Mitophagy in lung cancer

Mitochondria play a crucial role in the intracellular energy metabolism that is particularly important for tumor cells due to their high energy demands. Studies have shown that tumors can survive in the harsh tumor microenvironment by reducing mitophagy and increasing biogenesis in response to nutritional stress (Vazquez et al., 2013). This phenomenon has been supported by animal studies, where Li et al. (2018) found that mice lacking PINK1 and PRKN genes were more susceptible to developing KRAS-driven tumors. Interestingly, PINK1 has been identified as a molecular regulator of tumor aggressiveness and patient survival time in human transcriptome sequencing (Dai et al., 2019). Research shows that depleting PINK1 expression leads to reduced value-added and increased cell death in non-small cell lung cancer. This finding was also observed in liver cancer, where researchers noted that increased PINK1 expression in liver cancer bound TP53 and inhibited the tumor suppressor function of TP53. Conversely, decreased PINK1 expression allowed more TP53 to enter the nucleus to express oncogenes, effectively controlling the tumor (Liu et al., 2017).

It is interesting to note that mitophagy adapter proteins BNIP3 and BNIP3L/NIX also play important roles in tumorigenesis through the PINK1/PRKN-independent mitophagy pathway. Studies have shown that BNIP3 and BNIP3L/NIX can lead to a shift in breast cancer cell metabolism from general cellular respiration to glycolysis, promoting tumor development through a Warburg effect (Chourasia et al., 2015), which was observed in both BNIP3L/NIX (Drake et al., 2017). In KRAS-mutated pancreatic cancer cells, BNIP3L/NIX has been shown to reduce ROS production by promoting mitophagy, enhancing mitochondrial redox stability, and allowing intracellular glucose banding to shift to glycolysis, thus promoting cancer cell growth (Humpton et al., 2019). However, in pancreatic ductal adenocarcinoma (PDAC) patients, BNIP3L/NIX mRNA expression was significantly correlated with reduced patient survival in the TCGA database. Although there is no identified basis for BNIP3L/NIX as a cancer treatment target in BNIP3L/NIX investigations, studies have reported that BNIP3L/NIX may be a prognostic marker for acute myeloid leukemia (AML) and be involved in determining the appropriateness of AML patients for mitochondria-targeted medication therapy. The mutation rate of the KRAS gene is high in non-small cell lung cancer, and targeted drugs for KRAS mutation are limited and often perform poorly (Hirsch et al., 2017), resulting in a low 5-year survival rate for lung cancer patients with KRAS mutation. Although mitophagy has not been directly studied in lung cancer, both KRAS and TP53 are key genes in lung cancer, and their mutations are associated with poor prognosis. Autophagy is closely related to lung cancer (Guo et al., 2011; Guo et al., 2013), and the spearhead also points to KRAS. Mitophagy, as a part of autophagy, must play a crucial role. Finally, the effect of mitophagy on tumors appears to be two-sided, perhaps related to the stage of tumor development, inhibiting tumor development in the early stages and promoting it in the advanced stages.

### 4.2 Mitochondrial dynamics in lung cancer

In cancer cells, altered dynamics of mitochondrial fission and fusion contribute to their adaptation to cellular stress and invasion, leading to resistance to chemotherapy or targeted drugs. Han et al. (2019) reported that under hypoxic conditions, ovarian cancer cells increased mitochondrial fission resulting in resistance to cisplatin. DRP1, plays a key role in this process as the initiator protein that controls mitochondrial fission and begins the fission process by binding to the outer mitochondrial membrane (Giacomello et al., 2020). In breast cancer, it found that DRP1-dependent mitochondrial fission was involved in metastasis and cancer cell resistance to the chemotherapeutic drug cisplatin. Similarly, in lung cancer, high expression of DRP1 had a positive effect on the invasive metastasis of cancer cells (Han et al., 2015). However, it has also been reported that allowing DRP1 expression in lung cancer can be implicated as a mechanism for lung carcinogenesis, and during lung cancer progression, there is a sustained decrease in DRP1 (Kim et al., 2018). Thus, it is important to strike a balance in mitochondrial fission, as both increased and decreased levels of DRP1 have been implicated in cancer development and progression. The development of the majority of cancer cells is underpinned by defective cell cycle checkpoints and failure in regulating apoptosis. Further studies on DRP1 suggest its involvement in apoptosis, with Bcl-2-associated X protein (BAX) being an important apoptosis-related protein regulating this process. Andreas Jenner et al. found that DRP1 can physically interact with the N-terminal region of BAX and promote apoptosis (Jenner et al., 2022). In hepatocellular carcinoma (HCC), researchers have discovered that decreased expression of mitochondrial fusion-related protein MFN1 inhibits mitochondria-dependent apoptosis, resulting in increased cancer progression (Huang et al., 2016). In addition, the intimate relationship between DRP1 and actin suggests the possibility of a connection between DRP1 and cell migration, specifically cancer cell metastasis. Studies have shown that in the breast, DRP1 expression is higher in invasive breast cancers than in non-invasive breast cancers, while the opposite is true for MFN1 expression. Silencing DRP1 or overexpressing MFN1 inhibits the formation of cellular lamellipodium, which reduces the ability of cancer cells to migrate (Zhao et al., 2013). Similar results have been observed in neurogenic tumors (Yin et al., 2016). Although not yet reported in lung cancer, these findings provide new ideas for medical treatments that could inhibit tumor metastasis.

### 4.3 Mitochondrial biogenesis in lung cancer

Multiple complex mechanisms, including genetic mutations, epigenetic modifications, and metabolic reprogramming, contribute to the impairment of mitochondrial biogenesis in lung cancer. In particular, oncogenic pathways like MAPK/ERK and PI3K/AKT/mTOR, which are frequently upregulated in lung cancer, have been shown to be involved in the regulation of mitochondrial function and biogenesis (Porporato et al., 2018). Studies have also revealed that key regulators of mitochondrial biogenesis such as PGC-1α, may play important roles in the pathogenesis of lung cancer. It showed that sirtuin 1 (SIRT1) can deacetylate PGC-1α to affect mitochondrial function and biogenesis, which has a role in hypoxia-induced chemoresistance in non-small cell lung cancer (NSCLC) (Xu R. et al., 2021). Short-term cisplatin exposure induces a ROS-mediated metabolic reprogramming, characterized by PGC-1α activation and increased mitochondrial mass, which can be counteracted by oxidative phosphorylation (OXPHOS) inhibition (Cruz-Bermúdez et al., 2019).

## 5 IPF

IPF is one of the most difficult respiratory diseases to treat, and its incidence is increasing year by year, primarily in middle-aged and elderly people after the age of 50. The disease is not obvious in its early manifestations and has a very poor prognosis after a clear diagnosis (Hutchinson et al., 2015), which is primarily attributable to its uncontrollable pulmonary fibrosis. IPF patients have a high incidence of pulmonary hypertension, lung cancer, and a number of cardiovascular disorders, which substantially complicates clinical therapy (Caminati et al., 2019). In IPF cells, researchers identified senescent cell characteristics such as telomere wear and oxidative stress (van Deursen, 2014), confirming that pulmonary fibrosis has a significant link with cellular senescence. Schafer et al. (2017) discovered that biomarkers associated with cellular senescence were detectable in IPF tissues, that the expression of cyclin-dependent kinase inhibitor 2A (CDKN2A), a marker of cellular senescence, increased with the severity of fibrosis, and that low expression of CDKN2A contributed to the restoration of healthy lungs in IPF mice. From this, we can infer that cellular senescence promotes the occurrence of pulmonary fibrosis to a certain extent, and the study of the mechanisms related to cellular senescence can help the exploration of IPF treatment.

Mitochondria play an important role in multiple cellular senescence mechanisms (van Deursen, 2014), and in senescent cells we can observe changes in mitochondria, including morphological changes such as increase in size, loss of mitochondrial cristae, and accumulation of mitochondrial ROS, with mutations in mtDNA, collectively known as senescence-associated mitochondrial dysfunction (SAMD). These alterations impair mitochondrial activity, and researchers have recently detected mitochondrial malfunction in macrophages, alveolar epithelial cells, and fibroblasts in the lungs of IPF patients (Mora et al., 2017) (Table 1).

**TABLE 1:** Mitochondrial quality control in IFP.

Molecules related to mitochondrial quality control	Regulation of pathways/Biological processes	Result	References
PINK1/PRKN	Mitochondrial fusion-promoting factor	Mitochondrial morphology change and increasing mitochondrial activity	Rana et al. (2013)
	DCT1-mediated mitophagy	Cellular senescence	Palikaras et al. (2015)
	PDGFR/PI3K/AKT signaling	Fibroblast proliferation and increasing pulmonary fibrosis	Kobayashi et al. (2016); Kurita et al. (2017)
	Impairment of mitophagy	Accumulation of abnormal mitochondria in alveolar epithelial cells and possible increase in fibrosis	Bueno et al. (2015)
	Regulation of ATF3 and integrated stress response (ISR)	Abnormal mitochondrial accumulation and elevating ROS	Bueno et al. (2018)
	mtDNA mutation, TGF-β secretion	Increased probability of fibrosis in the lungs and increased fibrosis	Ryu et al. (2017); Cheresh et al. (2015); Bueno et al. (2019)
MFN1/2	Mitochondrial fusion	AEC2 cell injury and fibrotic remodeling in the lung	Chung et al. (2019)
	Regulating cellular lipid metabolism		
NOX4	Mitochondrial ROS production	Lung macrophage profibrotic polarization and fibrotic repair	He et al. (2019)
	Mitochondrial biogenesis		
PCG-1α	Mitochondrial calcium uniporter (MCU)	Macrophage metabolic reprogramming related fibrotic repair	Gu et al. (2019)
	Thyroid hormone	Mitochondria-regulated apoptosis and fibrosis	Yu et al. (2018)
	Mitochondrial metabolism		

### 5.1 Mitophagy in IPF

Inadequate mitophagy is connected with a variety of degenerative diseases, most notably Parkinson’s disease, caused by the buildup of aberrant mitochondria. Researchers have extended the lifetime of *Drosophila melanogaster* by upregulating the PRKN gene, since this is connected with increased mitochondrial autophagic activity, which reduces the proteotoxicity of aberrant mitochondria that accumulate in cells (Rana et al., 2013). Not coincidentally, Palikaras et al. (2015) also found that upregulation of PINK1 or PRKN gene expression prolonged the lifespan of *Caenorhabditis elegans*. In normal cells, these modifications may serve as a protective mechanism to eliminate defective mitochondria, reduce cell damage caused by abnormal mitochondria, achieve mitochondrial quality control, and lengthen cellular lifespan. In contrast, mitophagy dysregulation can be a crucial beginning factor for age-related illnesses. By knocking out the PRKN gene in mice with bleomycin-induced pulmonary fibrosis, Kenji Kobayashi et al. demonstrated that insufficient mitophagy-mediated PDGFR/PI3K/AKT activation is a potential cause of fibroblast (the primary pathogenic cell in IPF) appreciation. In these mice, insufficient PDGFR/PI3K/AKT activation is primarily due to reduced PRKN expression (Kobayashi et al., 2016). Meanwhile, pirfenidone (PFD) is the main drug for the treatment of pulmonary fibrosis, and preventing the development of fibrosis and scarring. Kurita et al. (2017) discovered a new mechanism for its treatment of IPF: PFD can induce both autophagy and mitophagy, a process that is achieved by enhancing PRKN expression. And this process is involved in the inhibition of fibroblast differentiation. It is concluded that PFD inhibits PARIN knockdown-induced myofibroblast differentiation by reducing mitochondrial ROS and PDGFR-PI3K-AKT activation. And PINK1, as a crucial link in the conventional process of mitophagy, also plays a crucial role in the pathogenesis of IPF, Bueno et al. (2015) discovered a significant decrease in PINK1 expression in alveolar epithelial cells of mice with pulmonary fibrosis, which resulted in the inability of mitochondria to recruit sufficient PRKN and a deficiency in mitophagy. This explains why numerous mitochondria with abnormal morphology and function were discovered in alveolar epithelial cells of IPF patients. In addition, if PINK1 expression is downregulated in young mice, similar morphologically and functionally abnormal mitochondria are found in their alveolar epithelial cells, and these mice are more susceptible to lung fibrosis. Endoplasmic reticulum stress is essential to the pathophysiology of pulmonary IPF (Phan et al., 2021) and researchers have shown that PINK1 is a major contributor to the high levels of endoplasmic reticulum stress in IPF (Lawson et al., 2011; Bueno et al., 2018). It was discovered that activating transcription factor 3 (ATF3), a component of the integrated stress response (ISR), negatively regulates the PINK1 gene and that cells that overexpress ATF3 have an abundance of aberrant mitochondria and high ROS levels. Importantly, the researchers also discovered that ATF3 expression in alveolar epithelial cells increases with age, which correlates implicitly with the age-related correlation of IPF (Bueno et al., 2018). Statistical studies have shown that mtDNA levels in bronchoalveolar lavage fluid (BALF) and plasma of IPF patients are a reliable predictor of prognosis (Ryu et al., 2017). In an asbestos-induced mouse model of pulmonary fibrosis, mtDNA-damaged mice are susceptible to pulmonary fibrosis (Cheresh et al., 2015). Similarly, mitophagy regulates the integrity of mtDNA, and a study by Bueno et al. (2019) discovered that mutation and release of mtDNA in IPF are dependent on the deletion of PINK1, whereas loss of PINK1 results in the secretion of the pro-fibrotic factor TGF-β. In conclusion, the PINK1/PRKN pathway is significant in IPF caused by defective mitophagy, and related research is anticipated to pave the door for a new therapeutic strategy for IPF.

### 5.2 Mitochondrial dynamics in IPF

Alveolar epithelial cells are important therapeutic cells in the pathogenesis of IPF, and researchers have observed enlarged mitochondria in such cells in IPF (Bueno et al., 2015), and single-cell sequencing has also suggested elevated MFN2 mRNA expression in IPF (Xu et al., 2016). In addition, Kuei-Pin Chung et al. discovered loss of the MFN1 and MFN2 in murine alveolar type 2 epithelial (AEC2) cells leads to morbidity and mortality associated with spontaneous lung fibrosis (Chung et al., 2019). Importantly, researchers have also revealed that MFN1/2 plays a crucial role in regulating cellular lipid metabolism. These findings highlight the significance of MFN1/2 in IPF and suggest its involvement in the regulation of cellular lipid metabolism and the development of pulmonary fibrosis. Notably, IPF is associated with complicated mitochondrial morphological abnormalities, including mitochondrial expansion and fragmentation, as well as modifications in mitochondrial cristae. OPA1, a crucial molecule that mediates the remodeling of mitochondrial cristae, merits investigation in IPF. Furthermore, the mitochondrial morphology of other cell types, such as lung macrophages and lung fibroblasts, has not been characterized in detail. However, it is plausible that these cell types undergo morphological changes in the context of IPF. It should be noted that mitochondrial homeostasis is disrupted in various aspects of cellular senescence (Vasileiou et al., 2019). Consequently, it would be logically inconsistent to solely focus on examining the kinetic function in IPF without considering the broader implications of mitochondrial dysfunction in cellular senescence as a whole. As stated previously, mitophagy controls kinetics, and the same kinetics is linked to other mitochondrial activities, such as mitochondrial membrane potential (MMP) changes, unfolded protein response, and ER stress. Future study should concentrate on determining how they interact in IPF.

### 5.3 Mitochondrial biogenesis in IPF

Repeated damage to type II alveolar epithelial cells is an important cause of pulmonary fibrosis, but repeated damage to such cells is not necessary in the progression of pulmonary fibrosis (Larson-Casey et al., 2019). Studies have shown that pulmonary macrophages are capable of producing large amounts of ROS as well as collagen synthesis substrates in pulmonary fibrosis, which is significant for the development of pulmonary fibrosis. He et al. (2019) found that NOX4 was high in the lung macrophages of asbestosis patients, and NOX4-deficient animals were protected from asbestos-induced pulmonary fibrosis. Furthermore, investigations demonstrated that in lung macrophages, NOX4 promoted macrophage pro-fibrotic polarization. Also, researchers have found evidence for the role of PCG-1α in macrophages and its association with the mitochondrial calcium uniporter (MCU) in pulmonary fibrosis. The elevated levels of PCG-1α in lung macrophages from IPF patients were observed. Notably, this elevation was identified only in lung macrophages expressing MCU, indicating a link between mitochondrial calcium metabolism and PCG-1α-mediated mitochondrial biogenesis (Gu et al., 2019). Yu et al. (2018) also discovered a correlation between the anti-fibrotic effect of thyroid hormones and PCG-1α-induced mitochondrial biogenesis. Although direct evidence of mitochondrial biogenesis and progression of pulmonary fibrosis illness has been discovered, the particular involvement of PCG- 1α in this remains to be studied, maybe in relation to reactive oxygen species or cellular senescence. Whether it is the negative effect of elevated NOX4 on pulmonary fibrosis or the fact that elevated PCG- 1α in IPF helps to reduce pulmonary fibrosis, it is suggesting that mitochondrial biogenesis has an effect on fibrosis and, paradoxically, that excess mitochondria produce excess ROS. As previously said, mitochondrial biogenesis cannot be considered a separate process because it is intimately tied to other mitochondrial physiological actions; nonetheless, their specific manifestations in the fibrosis process have yet to be investigated.

## 6 Conclusion

As research into mitochondrial quality control has progressed, researchers have identified important roles in disease development, particularly in neurodegenerative diseases such as Parkinson’s and cardiovascular disease, which have proved worth exploring (Chang et al., 2022; Eldeeb et al., 2022). In the respiratory system, researchers have focused on mitochondrial quality control in oxidative stress and cellular senescence. Molecules such as PINK1, PRKN, DRP1, and PCG1-α, as well as the signaling pathways they affect, have been shown to play an important role in respiratory-related diseases. However, conflicting reports regarding different pathological processes, such as the role of mitochondrial autophagy in lung cancer, pose difficulties in the study of targeted mitochondrial quality control drugs. There appears to be a delicate balance between the mitochondrial quality control processes in the physiological state. In COPD and lung cancer, mitochondrial quality control is often involved in disease development by influencing oxidative stress and apoptosis, and in IPF appears to be involved in the disease process by participating in the cellular senescence process, coinciding with the epidemiological features of the disease. In conclusion, this review describes recent research findings on mitochondrial quality control in lung diseases.
